# Supplementary material for: Targeted knockdown of ribulose-1, 5-bisphosphate carboxylase-oxygenase in rice mesophyll cells
Source: J Plant Physiol. 2021 May;260:153395. doi: 10.1016/j.jplph.2021.153395 (PMC8090977; doi:10.1016/j.jplph.2021.153395)
Supplement: Supplementary file 1 [file mmc1.docx]

(A) *rbcs2*-165


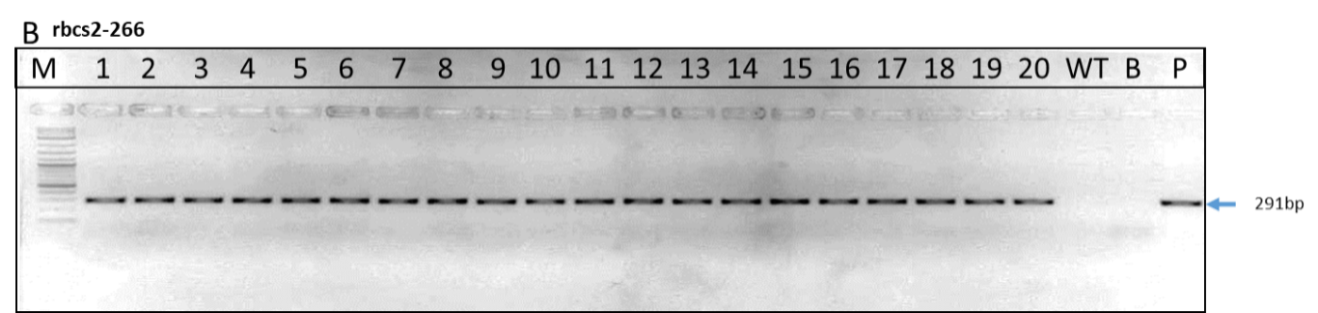

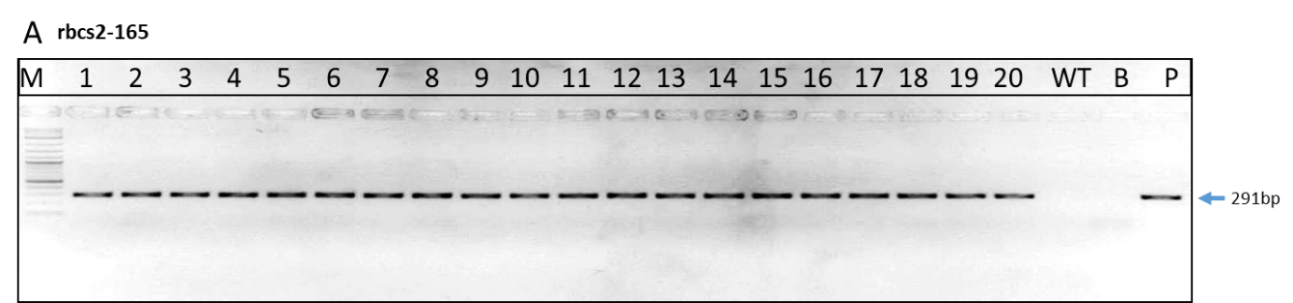

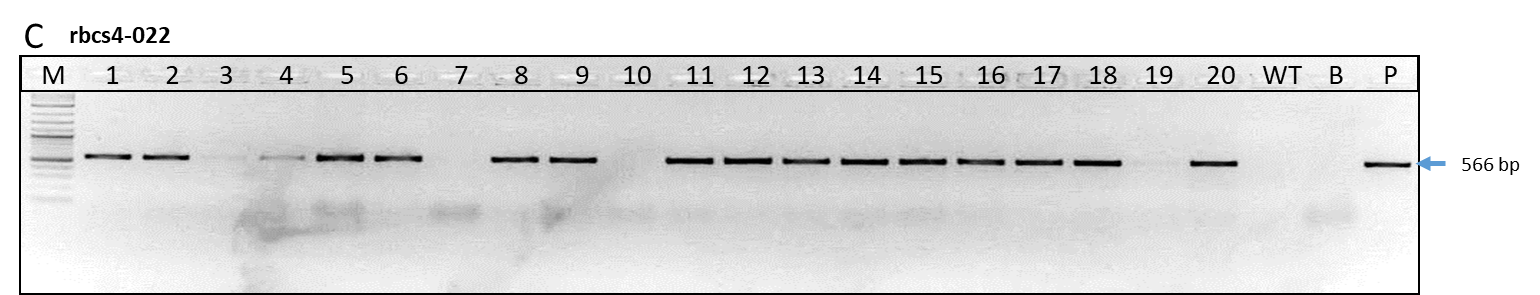

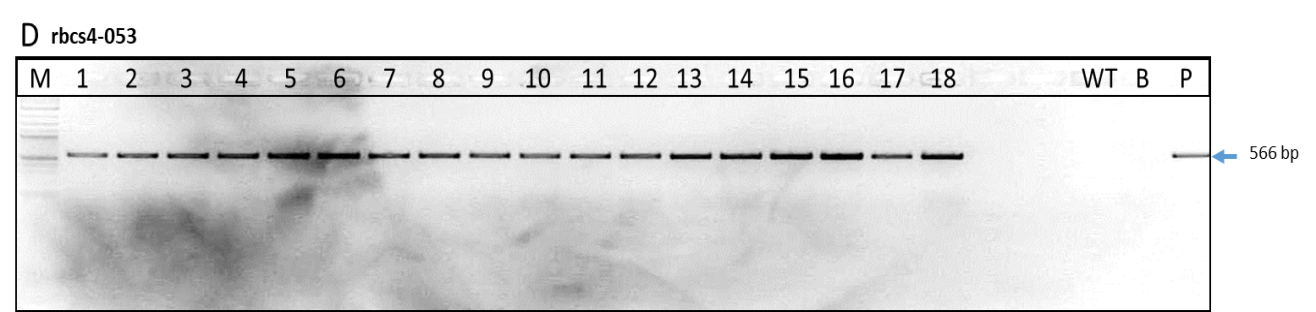


(B) *rbcs2*-266

(C) *rbcs4*-022

(D) *rbcs4*-053

**Fig. S1.** PCR analysis of *rbcs2* and *rbcs4* knockdown lines (numbers). Wild-type (WT) was used as a negative control and the vector plasmid (P) as positive control. B indicates blank (H_2_O), M indicates a ladder (100bp).

*rbcs2-*165

*rbcs2*-266

*rbcs4*-022

*rbcs4*-053


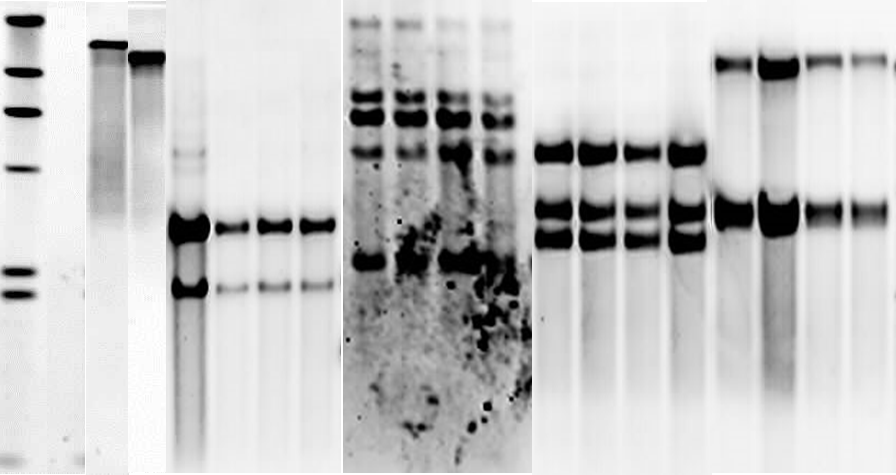


MW WT P_1_ P_2_ 1 2 3 4 1 2 3 4 1 2 3 4 1 2 3 4

4361

23130

2322

2017

9416

6557

**Fig. S2.** DNA blot analysis of *rbcs2* and *rbcs4* knockdown lines (numbers). The genomic DNA of each plant was extracted from leaves with the CTAB method and digested with *BgL*II. Wild-type plants (WT) were used as a negative control and the vector plasmid (P1 for antisense-*OsRBCS2* construct and P2 for antisense*-OsRBCS2* construct) as a positive control. A DIG-labeled DNA molecular marker indicates the molecular weight (MW).

BSC


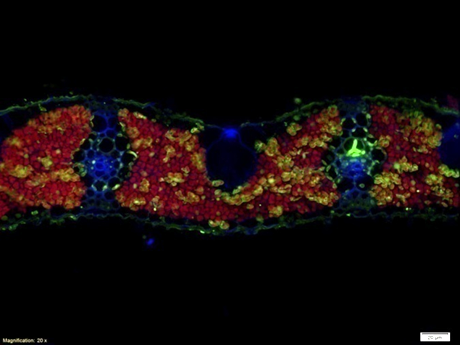


Wild-type


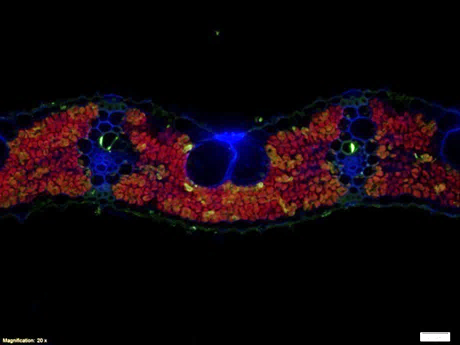


*rbcs2*-165


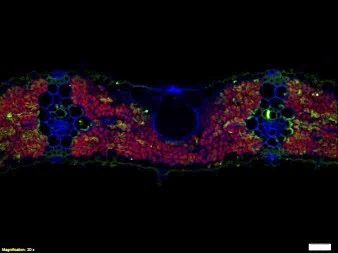


*rbcs2*-266


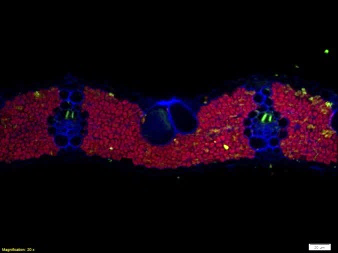


*rbcs4-0*22


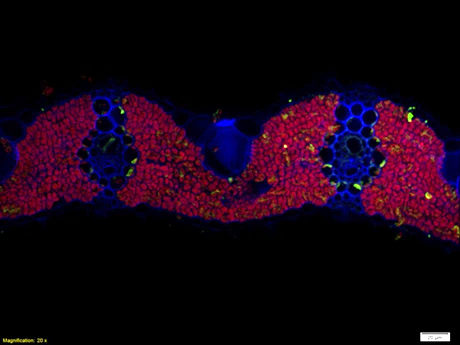


*rbcs4*-053

MC

**Fig. S3.** Representative images of immunolocalization of Rubisco protein in leaves from wild-type (WT), *rbcs2* and *rbcs4* knockdown lines. Anti-Rubisco rabbit polyclonal primary antibody diluted 1:200 plus Alexa Fluor 488 goat anti-rabbit IgG as secondary antibody diluted 1:200 was used to probe for rbcL (shown in green color). Chlorophyll is seen as a red autofluorescence. The cell wall was visualized by co-staining with calcofluor white and is shown in blue. Magnification: 200x. Scale bar: 20 μm. BSC: Bundle sheath cell. MC: mesophyll cell.


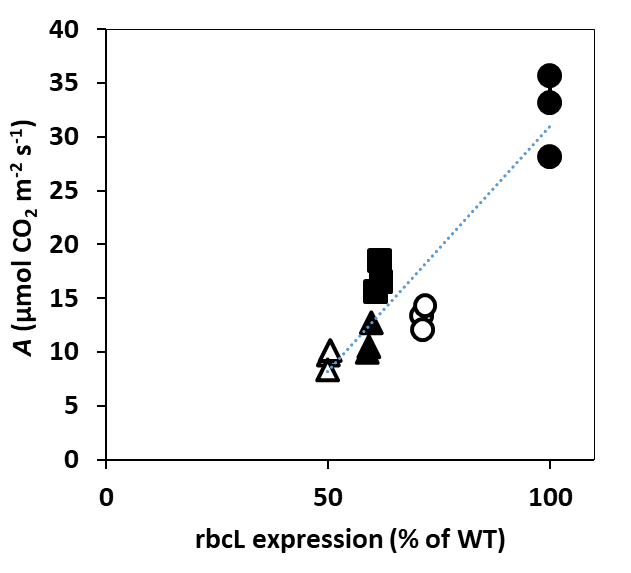


**Fig. S4.** Net CO_2_ assimilation rate (*A*) versus Rubisco large subunit protein content (% of WT). Gas exchange measurements were made at a light intensity of 2,000 µmol photons m^–2^ s^–1^ and at CO_2_ concentration of 400 µmol CO_2_ mol air^–1^. Black circles, white circles, black triangles, white triangles and black squares represent data from wild-type, *rbcs2*-165, *rbcs2*-226, *rbcs4*-022 and *rbcs4*-053. The rbcL expressions were measured from one leaf per plant. Values are expressed as the percentage protein abundance compared to the wild-type (WT). The same plant had been used for the measurements of rbcL expression and gas-exchange.


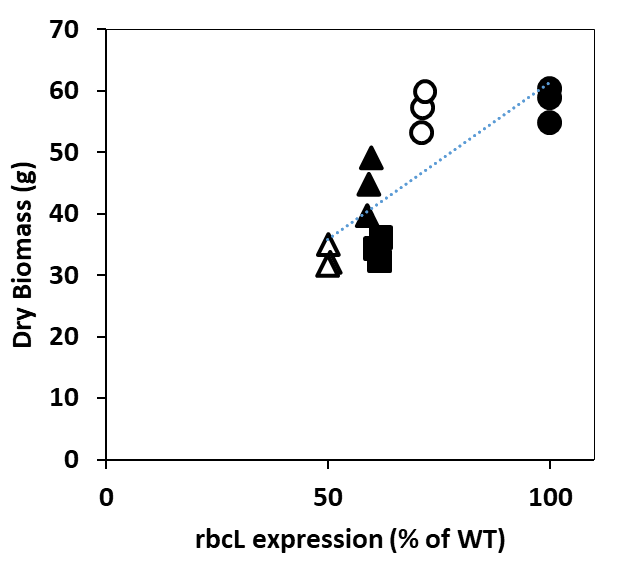


**A**


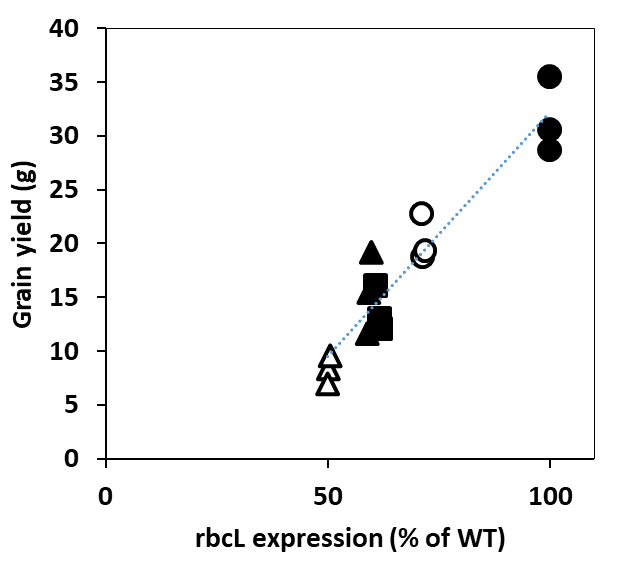


**B**

**Fig. S5.** Dry biomass (**A**) and grain yield (**B**) versus Rubisco large subunit protein content (% of WT). Biomass is the total dry weight of leaf, stem and sheath tissue. Black circles, white circles, black triangles, white triangles and black squares represent data from wild-type, *rbcs2*-165, *rbcs2*-226, *rbcs4*-022 and *rbcs4*-053. The rbcL expressions were measured from one leaf per plant. Values are expressed as the percentage protein abundance compared to the wild-type (WT). The same plant had been used for the measurements of rbcL expression, grain yield and dry biomass.
